# Significant Gene Biomarker Tyrosine Kinase Non-receptor 2 Mediated Cell Proliferation and Invasion in Colon Cancer

**DOI:** 10.3389/fgene.2021.653657

**Published:** 2021-08-04

**Authors:** Sunkai Ling, Yanru He, Xiaoxue Li, Yu Ma, Yuan Li, Bo Kong, Peilin Huang

**Affiliations:** ^1^Medical School of Southeast University, Nanjing, China; ^2^Department of Cardiology, Zhongda Hospital Affiliated to Southeast University, Nanjing, China; ^3^Department of Surgery, Klinikum rechts der Isar, School of Medicine, Technical University of Munich (TUM), Munich, Germany; ^4^Department of General Surgery, University of Ulm, Ulm, Germany

**Keywords:** colon cancer, TNK2, bioinformaticanalysis, miR-125a-3p, biomarker

## Abstract

**Objective:** This study aimed to investigate the expression and biological functions of *TNK2* and miR-125a-3p in colon cancer.

**Materials and methods:** The expression of *TNK2* and miR-125a-3p in colon cancer tissues was analyzed using data deposited on public databases including UALCAN and ONCOMINE. We verified their expression in colon cancer cell lines by RT-qPCR and western blotting. By regulating the expression of *TNK2* and miR-125a-3p in colon cancer cells, their functions and potential mechanisms were explored.

**Results:***TNK2* was overexpressed in colon cancer cell lines, and it was found to directly bind to miR-125a-3p, which was downregulated in these cell lines. Their expression affected the proliferation and invasion of colon cancer cells. Additionally, colon cancer patients with lower *TNK2* expression had better prognoses than those with higher *TNK2* expression.

**Conclusion:** Our results indicated that *TNK2* and miR-125a-3p play critical roles in colon cancer, and could also serve as biomarkers for the diagnosis and prognosis of this malignant disease.

## Introduction

Colon adenocarcinoma (COAD) is a malignancy with high morbidity and mortality. Moreover, it is the second most prevalent cancer in men and the third in women (Miller et al., 2019). COAD incidence is higher in men and is influenced by age, with a median age of approximately 70 years at the time of diagnosis in developed countries (Chung, 2018). Consequently, the cornerstones of therapy are surgery, neoadjuvant radiotherapy, and systemic treatment, including chemotherapy, targeted therapy, hormonal therapy, and immunotherapy (Segal and Saltz, 2009; Ilson, 2018; Wu, 2018). The prognosis of colon cancer is strongly associated with the therapeutic methods employed, which are based on the stage of the disease at diagnosis. Meanwhile, diagnosis at earlier stages indicates better therapy methods and lower mortality. Patients are often diagnosed at an advanced stage due to the lack of effective methods for early detection, which ultimately leads to particularly poor prognoses (Favoriti et al., 2016). Therefore, it is critical to explore the underlying molecular mechanisms involved and to develop more efficient means of diagnosing colon cancer.

Tyrosine kinase non-receptor 1 (TNK1) and TNK2 are members of the cytoplasmic tyrosine kinase family. TNK2, also known as ACK1 (activated CDC42-associated kinase) (Prieto-Echague and Miller, 2011), is a 120 kDa protein containing an N-terminal sterile alpha motif (SAM) domain, a kinase domain, an SH3 domain, and a Cdc42/Rac-interactive domain (CRIB) (Patricelli et al., 2011). The main function of TNK2 was initially identified as regulation of the cell cycle by binding to CDC42 (Manser et al., 1993). In addition, TNK2 can act as an effector of CDC42 to regulate cellular attachment and migration (Modzelewska et al., 2006). Also, TNK2 performed a momentous function in different cancers. In non-small cell lung cancer, TNK2 might affects the development of tumors by influencing the tumor immune microenvironment (Zhu et al., 2020). Moreover, TNK2 activation as a novel mechanism of EGFR inhibitor resistance is revealed for the guiding potential combined strategies (Zhang et al., 2020). In castration-resistant prostate cancer, TNK2 drives the malignant state via a feed-forward ACK1/pY88-H4/WDR5/MLL2/AR epigenetic circuit (Mahajan et al., 2017). Although a growing number of studies have reported TNK2 expression and its molecular mechanisms involved in different cancers, there is still not much to be understood of colon cancer in this context. Gene expression microarray and RNA sequencing technologies have been used to detect gene expression in cells and tissues, which helps researchers to explore potential targets and pathways associated with disease. In this study, we utilized a public database to screen for differential expression of TNK2 between caners and their corresponding non-cancerous tissues. Furthermore, bioinformatic analysis was used to evaluate the function of TNK2 in colon cancer, including co-expression of genes and promoter methylation levels. Kaplan–Meier survival analysis was considered as a prognostic index of TNK2 in colon cancer.

In this study, we found that the expression of TNK2 was upregulated in colon cancer cell lines and that it directly binds to miR-125a-3p, which is downregulated in colon cancer cells. The expression of TNK2 was affected by the binding of miR-125a-3p to the 3′-UTR of *TNK2*, and both were involved in regulating a pathway critical for the proliferation and invasion of colon cancer cells. In conclusion, our results suggested that TNK2 and miR-125a-3p might serve as potential diagnostic and therapeutic targets.

## Materials and Methods

### Cell Culture

Human colon cancer cell lines (LOVO, HT29, HCT116, and SW620) and the human colonic epithelial cell line (NCM460) were purchased from GeneChem (Shanghai, China). Cells were cultured in Dulbecco's modified Eagle's medium (DMEM) (Gibco, Thermo Fisher Scientific, Waltham, MA, USA) supplemented with 10% fetal bovine serum (FBS; Gibco, Thermo Fisher Scientific) and 1% penicillin and streptomycin (Solarbio, Beijing, China), and the cells were maintained in a 37°C incubator containing 5% CO_2_. The medium was replaced every 24–48 h according to the condition of the medium. Cell morphology and density were observed under an inverted microscope, and 0.25% trypsin (Gibco, Thermo Fisher Scientific) was used to digest cells for passaging when they reached 80% confluence.

### Quantitative Real-Time PCR (RT-qPCR)

TRIzol reagent (Takara Bio Inc., China) was used to isolate total RNA from colon cancer cells. RNA concentrations were measured and recorded using a spectrophotometer (Titertek-Berthold Colibri). Complementary DNA (cDNA) was synthesized using a PrimeScript RT reagent kit (Takara Bio Inc.), and RT-qPCR was performed using SYBR Premix Ex Taq (Takara Bio Inc.). The threshold cycle (Ct) values for *TNK2* were normalized against the Ct values of *GAPDH*, while miR-125a-3p was normalized against the Ct values for U6 snRNA. The relative expression values of RNAs were calculated using the 2^−Δ*ΔCt*^ method.

### Western Blot Analysis

A protein extraction kit (KeyGEN Biotech, Nanjing, China) was used to extract total proteins from colon cancer cells. Then, protein concentrations were quantified using a BCA Protein Assay kit (KeyGEN Biotech, Nanjing, China). After boiling for 5 min with loading buffer, each sample containing an equivalent amount of protein (20 μg) was separated by 10% sodium dodecyl sulfate-polyacrylamide gel electrophoresis (SDS-PAGE) and transferred to polyvinylidene difluoride (PVDF) membranes. After blocking with 5% skim milk powder for 1 h at room temperature, the PVDF membranes were incubated overnight at 4°C with a rabbit anti-TNK2 antibody (1:1,000) (Affinity, USA) and a rabbit monoclonal antibody against GAPDH (1:5,000) (Beyotime Biotechnology, Beijing, China). Then, the membranes were washed three times with TBS-T buffer, which was followed by incubation with a goat anti-rabbit secondary antibody (1:5,000, Beyotime Biotechnology) for one h at room temperature. Immunoreactive proteins were detected using an electrochemiluminescence (ECL) Reagent (Affinity, USA) and an automatic chemiluminescence image analysis system (Tanon 5200).

### RNA Interference

We used Lipofectamine 3000 (Invitrogen, USA) for small interfering RNA (siRNA) and siRNA mimic transfection. A siRNA targeting *TNK2* mRNA (si-*TNK2*) was designed and synthesized by GeneChem (Shanghai, China), and a miR-125a-3p mimic was designed and constructed by GenPharma (Shanghai, China). After transfection, RT-qPCR was performed to detect transfection efficiency.

### Colony Formation Assay

Cell culture dishes (35 mm) were used to cover 500 cells in 2 ml complete medium after transfection. The medium was replaced every 48–72 h to ensure sufficient nutrient supply. Cell colony clones were fixed with 4% paraformaldehyde for 1 min, stained with 0.1% crystal violet solution for half an hour after culturing for 14 days, and then imaged by a camera and counted using ImageJ.

### CCK-8 Assay

CCK-8 assays were performed to detect cell proliferation using an Enhanced Cell Counting Kit-8 (Beyotime Biotechnology). SW620 cells were seeded into 96-well plates and transfected after cell adherence. Then 100 μl complete medium with 10% CCK-8 solution was added to each well. After 2 h of incubation, the OD_450_ was measured using a microplate reader. We measured once a day for 5 consecutive days. This experiment was repeated three times.

### Dual-Luciferase Assay

Luciferase assays were performed using a Dual-Luciferase Reporter Assay System kit (E2920, Promega, USA). The wild-type (WT) *TNK2* 3′UTR and a mutant version incorporating the Renilla luciferase gene (*hRluc*) and firefly luciferase gene (*hLuc*) were obtained from GeneChem. 293T cells were co-transfected into cells with the miR-125a-3p mimic or NC mimic for 48 h. Firefly luciferase activity and Renilla luciferase activity were measured using a microplate reader. Firefly luciferase activity was normalized to Renilla luciferase activity to obtain relative values of luciferase expression.

### Transwell Invasion Assay

Transwell invasion assays were performed to detect cell invasion. The transwell chambers were coated with Matrigel (BD Biosciences, CA, USA), and the bottom chambers were added 500 μl of complete medium, for the upper chambers, SW620 cells were seeded with serum-free medium, each contains 4 × 10^4^ cells. After incubation for 24 h, the cells were fixed with 4% formaldehyde and then stained with 0.1% crystal violet solution. Images were collected by microscopy and the number of invasive cells was counted by ImageJ.

### Public Online Database

The Cancer Genome Atlas (TCGA) is a momentous cancer genomics program, and it includes 33 cancer types and over 2,000 primary cancer. Moreover, the data base also gathered over 2.5 petabytes of genomic, epigenomic, transcriptomic, and proteomic data. In this research, we collected clinical data and gene expression on 385 patients with colon cancer through TCGA.

The Human Protein Atlas (HPA) database has collected a valuable repository of transcriptome and proteome data, which supports researchers to study protein localization and expression in human tissues and cells (Thul and Lindskog, 2018). To validate the IHC results, TNK2 expression data in colon cancer and normal tissues was obtained from the HPA database. Immunohistochemical staining was detected and annotated by specially educated personnel, and the staining patterns were classified into high, medium, low, and not detected.

The ONCOMINE gene expression array dataset (https://www.oncomine.org/), a cancer microarray database and web-based data-mining platform, contains 65 gene expression datasets comprising nearly 48 million gene expression measurements from over 4,700 microarray experiments (Rhodes et al., 2004). Differential expression analyses were conducted to analyze *TNK2* mRNA levels in different cancers. The expression of *TNK2* in clinical cancer specimens was compared with that in adjacent normal tissues. Furthermore, we indexed genes co-expressed with *TNK2* in colon cancer.

The Cancer Cell Line Encyclopedia (CCLE) (https://www.broadinstitute.org/ccle) provides a rigorous framework for studying genetic variants, candidate targets, and small-molecule and biological therapeutics and to identify new marker-driven cancer dependencies (Ghandi et al., 2019). The expression of *TNK2* in different cancer cell lines was verified using the CCLE dataset.

UALCAN (http://ualcan.path.uab.edu) is an interactive web portal to perform in-depth analyses of The Cancer Genome Atlas (TCGA) gene expression data. UALCAN uses TCGA level 3 RNA-seq and clinical data from 31 cancer types (Chandrashekar et al., 2017). We used UALCAN to verify the expression of *TNK2* in different cancers and pathological stages; moreover, miRNA prediction and DNA methylation level data were obtained via this portal.

The LinkedOmics database (http://www.linkedomics.org) contains multi-omics and clinical data for 32 cancer types and 11,158 patients from TCGA (Vasaikar et al., 2018). It provides a unique platform for researchers to obtain, analyze, and compare multi-centric data regarding cancers. We used LinkedOmics to analyze prognostic values of *TNK2* levels in colon cancer and genes co-expressed with *TNK2*.

Gene Expression Profiling Interactive Analysis (GEPIA) is a newly developed web-based tool for analyzing RNA sequencing expression, with customizable functionality data based on TCGA and GTEx data (Tang et al., 2017). GEPIA is available at http://gepia.cancer-pku.cn/. We analyzed the prognostic value of identifying *TNK2* levels in patients with colon cancer.

EMBL-EBI (https://www.ebi.ac.uk/gxa/home) has become one of the largest metagenomic repositories in the world, provides a convenient platform for supporting the analysis of a range of study types, and is generated using a variety of different sequencing technologies (Mitchell et al., 2018). *TNK2* expression in colon cell lines was verified using the EMBL-EBI dataset.

### Statistical Analysis

Data between groups are presented as means ± standard deviation. Statistical significance between two groups was determined by Student's *t*-test, one-way ANOVA or two-way ANOVA followed by Tukey's test was performed to analyze when among multiple groups, Correlations between two groups were analyzed by the Pearson correlations. SPSS (v.13.0.0; SPSS Inc, USA) was used to calculate the significance. Kaplan-Meier plots relation analysis was used to generate the survival curve. ^*^P < 0.05 was considered statistically significant, and ^**^P < 0.01 was considered highly statistically significant.

## Results

### Bioinformatic Analysis of TNK2 Expression in Colon Cancer From Public Databases

**Figure 1:**
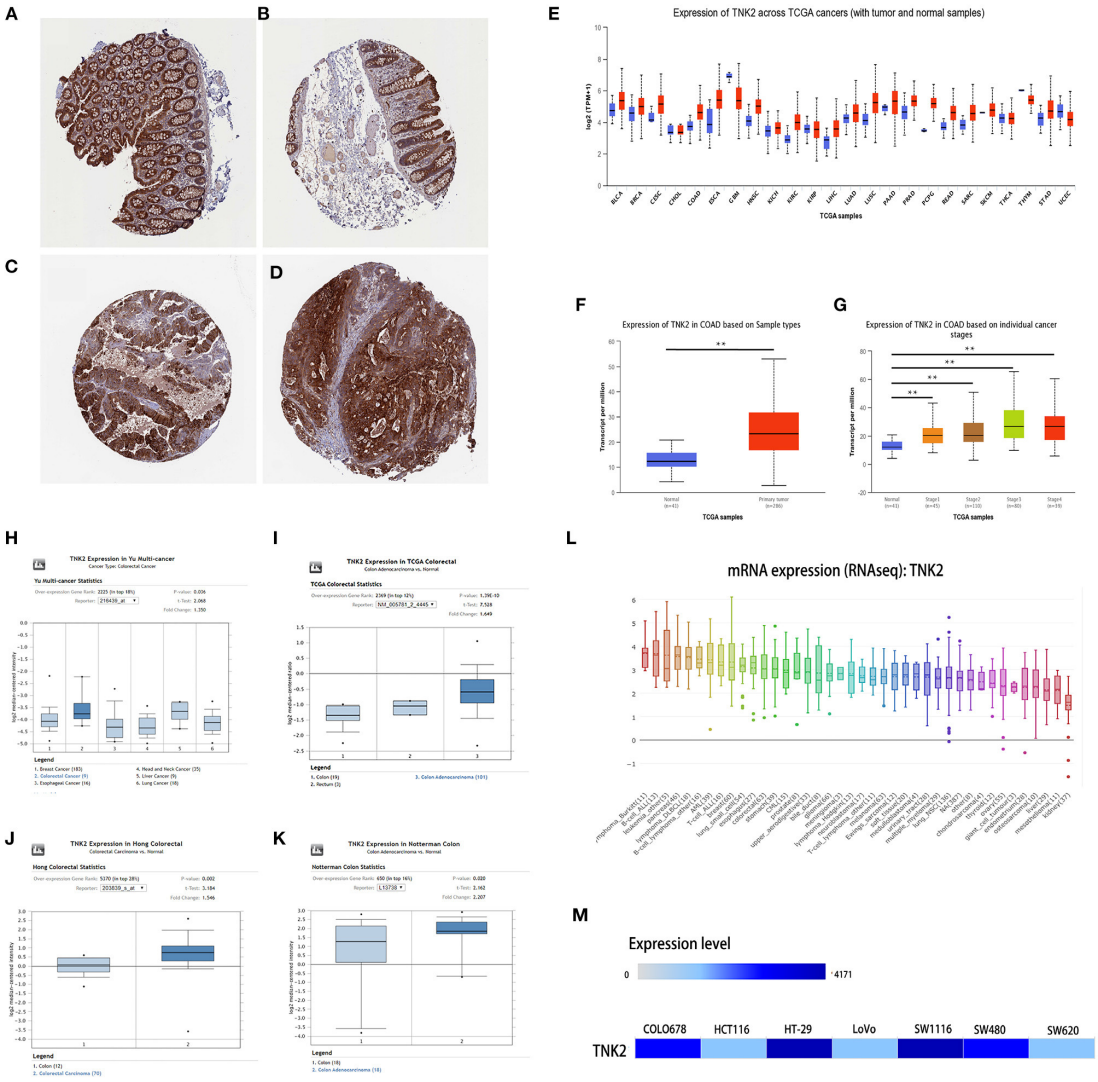
TNK2 was overexpressed in colon cancer tissues and cell lines from public databases. **(A,B)** Immunohistochemical staining revealed TNK2 expression in normal colon tissue samples **(C,D)** and cancer tissues samples analyzed in HPA data. **(E)** TNK2 expression in pan-cancer tissues and adjacent normal tissues, **(F)** TNK2 expression in colon cancer and normal tissues, **(G)** TNK2 expression at each stage of colon cancer. **(H–K)** TNK2 expression in Yu Multi-cancer Statistics, TCGA Colorectal Statistics, Hong Colorectal Statistics, and Notterman Colon Statistics. **(L)** Expression of TNK2 in different cell lines and analyzed by CCLE. **(M)** Expression of TNK2 in colon cell lines and analyzed using EMBL-EBI data. **p < 0.01.

According to immunohistochemical data from the HPA database, staining revealed that TNK2 exhibited low expression in endothelial cells in normal colon tissue samples (Figures 1A,B) and high expression in malignant cells in colon cancer tissues (Figures 1C,D). Immunohistochemical staining revealed TNK2 expression in colon cancer tissues was more pronounced than that in normal colon tissues.

TNK2 expression across 24 cancer types in TCGA data was analyzed by UALCAN, independent-samples *T* test was used to analyze the significant differences between different cancers and their adjacent normal tissues, and the result shows that TNK2 expression was universally higher in most types of cancers, including breast cancer, COAD, and pancreatic cancer (Figure 1E). We further evaluated the expression of TNK2 in colon cancer and paired normal tissues at different tumor stages. A box plot (Figure 1F) illustrates that TNK2 expression was remarkably higher in colon cancer tissues than in adjacent normal tissues, and TNK2 was overexpressed at different stages of tumor progression (Figure 1G). We chose four statistics from the ONCOMINE dataset based on keyword searching. The keywords are as follows: Gene: TNK2, Analysis Type: Colorectal Cancer vs. Normal, and Differential Analysis. All data were calculated using a student's *t* test for the significant differences. Yu multi-cancer statistics showed that TNK2 expression in colorectal cancer is higher than that in breast, liver, esophageal, lung, and head and neck cancers (Figure 1H). TCGA colorectal cancer statistics indicated that TNK2 expression in malignant colon tissues was higher than that in normal tissues (Figure 1I). The same result was verified in Hong colorectal statistics and Notterman colon statistics (Figures 1J,K). We then used CCLE and EMBL-EBI to analyze TNK2 expression in cancer cell lines. When compared with different cancer cell lines, TNK2 was highly expressed in colorectal cancer cell lines from CCLE (Figure 1L). Moreover, EMBL-EBI data were also used to verify the expression of TNK2 translational factors in different colon cancer cell lines, and the results indicated that TNK2 levels were increased in most colon cancer cell lines when compared with the normal colon cell line (Figure 1M). A total of 385 patients with primary COAD from TCGA database were obtained to analyze the association between TNK2 expression and clinicopathologic features (Table 1).

**Table 1:** Association between TNK2 expression and clinicopathologic features.

	**TNK2 expression**		
	**High**	**Low**	**Total**
**Characteristic**	**(** ***n*** **=** **193)**	**(** ***n*** **=** **192)**	**(** ***n*** **=** **385)**
**Gender**			
Female	93 (48.2%)	88 (45.8%)	181 (47.0%)
Male	100 (51.8%)	104 (54.2)	204 (53.0%)
**Age**			
Mean (SD)	67.3 (13.1)	66.6 (12.5)	67.0 (12.8)
Median (Min, Max)	68[31,90]	68.5[36,90]	68[31,90]
**Stage**			
Stage I	27 (14.0%)	39 (20.3%)	66 (17.1%)
Stage II	12 (6.2%)	16 (0.83%)	28 (7.3%)
Stage IIA	47 (24.4%)	68 (35.4%)	115 (30.0%)
Stage IIB	4 (2.1%)	3 (1.6%)	7 (1.8%)
Stage IIC	NA	1 (0.5%)	1 (0.3%)
Stage III	13 (6.7%)	3 (1.6%)	16 (4.2%)
Stage IIIA	4 (2.1%)	1 (0.5%)	5 (1.3%)
Stage IIIB	27 (14.0%)	21 (10.9%)	48 (12.5%)
Stage IIIC	21 (10.9%)	14 (7.3%)	35 (9.1%)
Stage IV	24 (12.4%)	14 (7.3%)	38 (9.9%)
Stage IVA	8 (4.1%)	6 (3.1%)	14 (3.6%)
Stage IVB	1 (0.5%)	NA	1 (0.03%)
**Race**			
White	85 (44.0%)	96 (50.0%)	181 (47.0%)
Asia	4 (2.1%)	5 (2.6%)	9 (2.3%)
African	33 (17.1%)	19 (9.9%)	52 (13.5%)

### Overexpression of *TNK2* in Colon Cancer Cell Lines Involves Direct miR-125a-3p Binding

**Figure 2:**
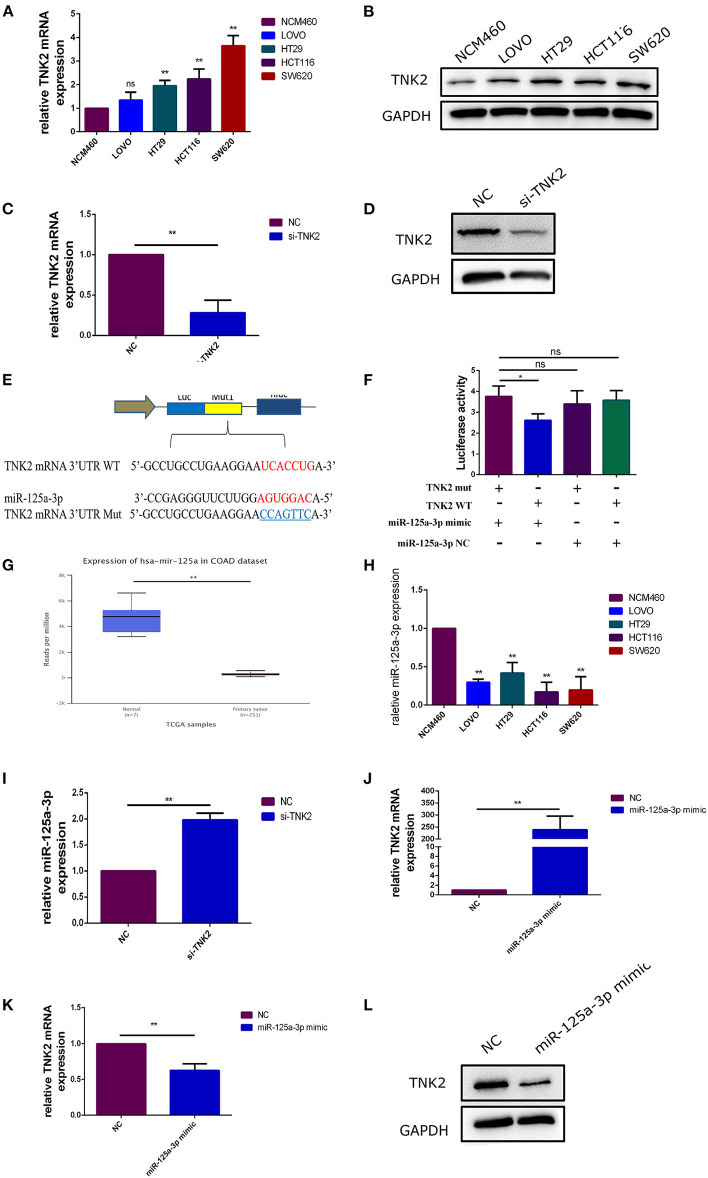
TNK2 was overexpressed and directly binds to miR-125a-3p in colon cancer cells. **(A,B)** Relative expression of TNK2 in COAD and normal colon cells, measured by RT-qPCR and western blot. **(C,D)** Relative expression of TNK2 after transfection of SW620 cells was measured by RT-qPCR. **(E)** Putative binding sites involving the TNK2 3′UTR and miR-125a-3p were predicted by miRBase. **(F)** Dual-luciferase reporter assays were performed to test putative binding sites in TNK2 3′UTR and miR-125a-3p. **(G)** Expression levels of miR-125a-3p in COAD and normal colon tissue, analyzed using UALCAN. **(H)** Relative expression of miR-125a-3p in COAD and normal colon cells, as measured by RT-qPCR. **(I,J)** Relative miR-125a-3p expression after transfection of SW620 cells was measured by RT-qPCR. **(K,L)** Relative TNK2 mRNA and protein expression were detected by RT-qPCR and western blotting after transfection. *p < 0.05, **p < 0.01.

RT-qPCR and western blotting were performed to investigate the expression of TNK2 in colon cancer cells. We then confirmed that TNK2 expression levels were significantly upregulated in COAD cell lines compared to those in NCM460 cells (Figures 2A,B). We then chose SW620 as an experimental cell line because the expression of TNK2 was highest in SW620 cells in comparison to the other cell lines. Si-*TNK2* was used to knock down *TNK2* expression in SW620 cells to investigate the function of TNK2 in COAD. After transfection for 48 h, RT-qPCR and WB were performed to evaluate transfection efficiency. Compared with the negative control (NC) group, the expression of TNK2 was significantly decreased in the si-*TNK2* group (Figures 2C,D). Using miRBase (http://www.mirbase.org/), we identified that *TNK2* 3′UTR might directly bind to miR-125a-3p (Figure 2E). Luciferase reporter assays revealed that the *TNK2* 3-UTR was strongly linked to miR-125a-3p (Figure 2F). In analyzing TCGA data by UALCAN, expression of miR-125a-3p was downregulated in COAD (Figure 2G). RT-qPCR was used to detect the expression of miR-125a-3p in colon cancer cell lines and in normal colon cells. The results showed that the expression of miR-125a-3p in colon cancer cells was significantly decreased compared to that in normal NCM460 cells (Figure 2H). To confirm the correlation between *TNK2* and miR-125a-3p, RT-qPCR was performed to detect the expression levels of miR-125a-3p after cells were transfected with si-*TNK2* or miR-125a-3p mimic. The results indicated that knockdown of *TNK2* expression upregulated the expression of miR-125a-3p (Figure 2I). The transfection efficiency of miR-125a-3p mimic was verified by RT-qPCR (Figure 2J). We found that upregulated expression of miR-125a-3p could reduce the expression of TNK2 at both the mRNA and protein levels (Figures 2K,L).

### *TNK2* and miR-125a-3p Play Vital Roles in Regulating Biological Function in SW620 Cells

**Figure 3:**
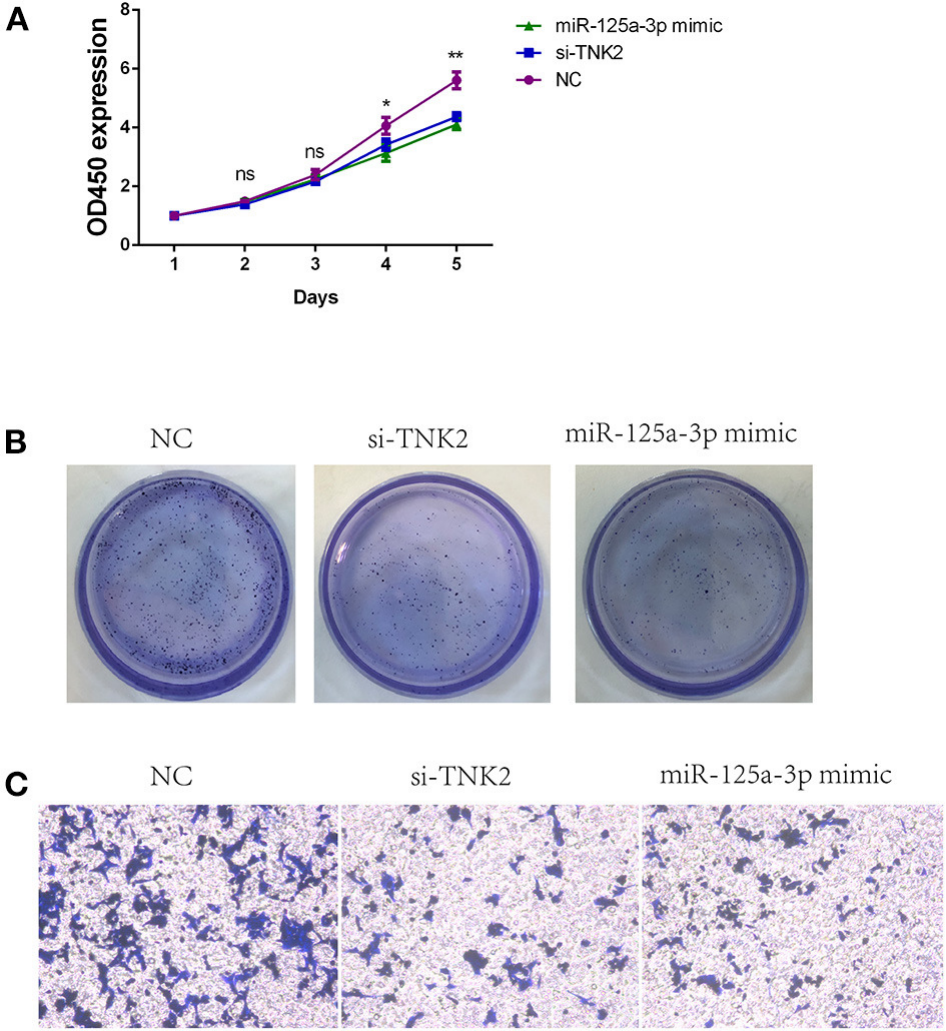
*TNK2* and miR-125a-3p are both crucial for regulating biological functions of PANC-1 cells. **(A)** Cell Counting Kit-8 assays were used to detect cell proliferation of SW620 cells after transfection. **(B)** Colony formation assays were used to detect clonogenic capacity in SW620 cells after transfection. **(C)** Transwell assays were employed to detect cell invasion ability in SW620 cells after transfection. *p < 0.05, **p < 0.01.

Knockdown of TNK2 and upregulation of miR-125a-3p expression levels could result in suppressed cell proliferation (Figure 3A) and inhibit invasiveness relative to the NC group (Figure 3B). Additionally, decreased *TNK2* and miR-125a-3p mimic inhibited the colony-forming capacity of SW620 cells (Figure 3C).

### Prognostic Value of TNK2 in Colon Cancer

**Figure 4:**
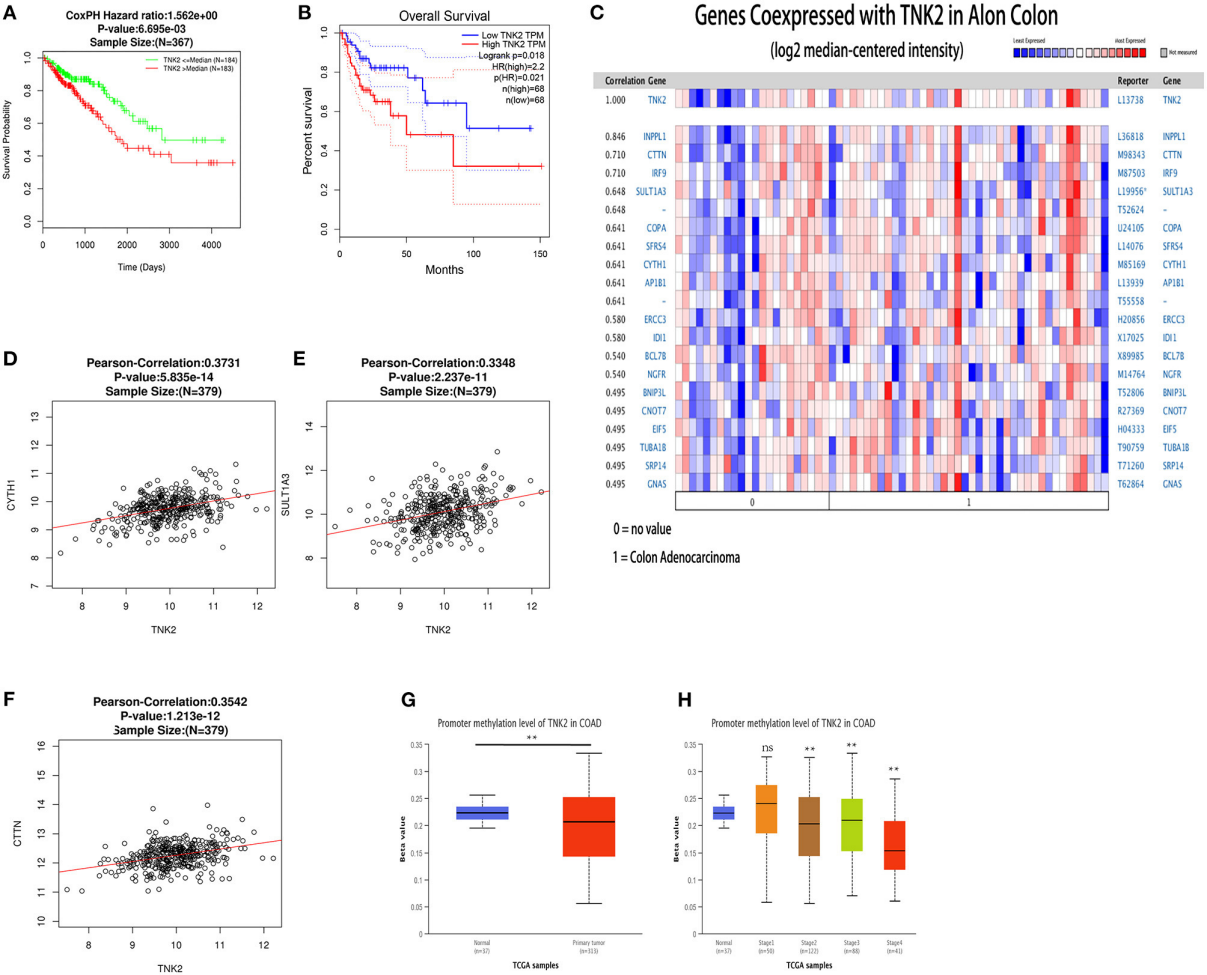
Prognostic value, co-expressed genes, and promoter methylation levels surrounding TNK2 expression in colon cancer patients. **(A)** Prognostic value of *TNK2* mRNA levels in colon cancer patients, as analyzed using LinkedOmics. **(B)** Prognostic value of *TNK2* mRNA levels in colon cancer patients, as analyzed using GEPIA. **(C)** Genes co-expressed with TNK2 in colon cancer, as analyzed using ONCOMINE. **(D–F)** Corrections involving *TNK2* and *SULT1A3, CYTH1*, and *CTTN* abundance in colon cancer, as analyzed using LinkedOmics. **(G)** *TNK2* promoter methylation levels in colon cancer cells compared with normal tissues. **(H)** *TNK2* promoter methylation levels at different colon cancer stages, as analyzed using UALCAN. **p < 0.01.

We investigated the prognostic value of TNK2 using GEPIA and LinkedOmics databases in colon cancer. The use of both datasets achieved a unified result; Kaplan-Meier survival analysis revealed that colon cancer patients with lower TNK2 expression had better prognoses than those with higher TNK2 expression (Figures 4A,B). Hence, the high expression of TNK2 was a prognostic factor for colon cancer.

### Genes Co-expressed With *TNK2* in Colon Cancer

Genes co-expressed with *TNK2* were analyzed using the Alon Colon statistic from ONCOMINE. We found that TNK2 expression was positively correlated with *INPPL1, CTTN, IRF9, SULT1A3, COPA, SFRS4, CYTH1*, and *AP1B1* (Figure 4C). We then identified the top ten co-expressed genes in LinkedOmics, three of which were verified to be significantly related by the Pearson correlations, including *CYTH1* (Figure 4D, *R* = 0.3731, *P* < 0.01), *SULT1A3* (Figure 4E, *R* = 0.3348, *P* < 0.01), and *CTTN* (Figure 4F, *R* = 0.3542, *P* < 0.01).

### Levels of *TNK2* Promoter Methylation in Colon Cancer

Using analysis of DNA methylation data from UALCAN, we verified that the *TNK2* promoter methylation level was reduced in colon cancer compared with that in normal tissues (Figure 4G), which was the opposite of *TNK2* expression. In stages 2, 3, and 4 of colon cancer, *TNK2* promoter methylation levels were also reduced (Figure 4H). Hence, these results revealed that the extent of *TNK2* promoter demethylation might cause the high TNK2 expression in colon cancer.

## Discussion

With the development of microarray and high-throughput sequencing technologies, thousands of genes have been detected as being implicated in different diseases. In the present study, we used microarray and RNA sequencing data obtained from a public dataset to investigate TNK2 expression in colon cancer. In our study, we verified that the expression of *TNK2* is upregulated in colon cancer cells compared with normal colon cells, and it was found to directly bind to miR-125a-3p, which was downregulated in such cell lines. Their expression can affect the proliferation and invasion of colon cancer cells. Additionally, Kaplan-Meier survival analysis revealed that the TNK2 expression was also prognostic relevant. We also identified genes co-expressed with *TNK2* in colon cancer. Because the data in each data set are derived from different ethnicities and at different times, we integrated analyses of multiple datasets and a public database in order to obtain more reliable results. We found that the elevated expression of TNK2 may be related to promoter methylation levels in colon cancer. Somehow, more experiments should be designed to verify this result in our further research. This study is the first to explore the relationships between *TNK2* and miR-125a-3p, and their potential mechanisms involved in colon cancer, which may benefit the diagnosis and treatment of colon cancer patients.

Previous studies have shown that TNK2 is a potential drug target in the treatment of metastatic CRC (Qi and Ding, 2018). In prostate cancer, TNK2 participates in a feed-forward epigenetic circuit involving ACK1/pY88-H4/WDR5/MLL2/AR and is necessary for malignant cancer (Mahajan et al., 2017). TNK2 is also reported to be a promising therapeutic target for the *PTPN11*-mutant (Jenkins et al., 2018). Recent research has demonstrated that TNK2 promotes EMT and cell migration and invasion by activating the AKT-POU2F1-ECD signaling pathway in GC cells (Xu et al., 2015). Another study suggested that TNK2 is an independent prognostic marker of HCC, which promotes cancer progression by downregulating WWOX and activating AKT signaling (Xie et al., 2015). Thus, TNK2 has been treated as a beneficial tumor promoter in many cancer types. In our study, the UALCAN and ONCOMINE datasets revealed that the expression of TNK2 is higher in colon cancer tissues than that in normal tissues. CCLE and EMBL-EBI databases also verified that TNK2 is highly expressed in colon cancer cell lines. We then performed RT-qPCR and WB to verify that the expression of TNK2 in colon cancer cell lines was higher than that in normal colon cells.

Recent studies have suggested that DNA methylation plays an important role in many diseases, including cancer, and cancer is often accompanied by abnormal DNA methylation, which is a key epigenetic process that is essential for the regulation of gene expression (Yang et al., 2017; Morgan et al., 2018). In the present report, UALCAN datasets were employed to identify *TNK2* promoter methylation levels in colon cancer cells. We presumed that high expression of TNK2 might be related to increased demethylation levels in colon cancer, in order to verify this assumption, we will design and perform some more experiments in the next study. TNK2 is crucial for PCSC survival (Mahajan et al., 2018). HCC patients with overexpression of TNK2 exhibit low survival rates (Wang et al., 2014). Moreover, TNK2 expression is independently associated with poor overall and relapse-free survival in non-small cell lung cancer (Tan et al., 2014). GEPIA and LinkedOmics datasets indicated that colon cancer patients with lower TNK2 expression had better prognoses than those with higher TNK2 expression. Although the expression levels of TNK2 were validated in colon cancer tissues, additional experiments are required in the future to confirm the expression and function of TNK2 in colon cancer. Among the genes identified as being co-expressed with *TNK2* in this study, *CYTH1* has been reported to be a major regulator of cell adhesion and engraftment in human HSPCs, and *CYTH1* defects seriously affect the mobility and localization of HSPCs (Rak et al., 2017). The function of CYTH1 in colon cancer is poorly understood, we hypothesized that TNK2 is possible to work in synergy with CYTH1 or other co-expressed proteins. For this part, we will verify it in our future research.

Taken together, we systemically demonstrated that TNK2 expression was increased in colon cancer and that such expression may be regulated by miR-125a-3p or *TNK2* promoter demethylation levels. Our results indicated that the expression of *TNK2* and miR-125a-3p affects proliferation and invasion by colon cancer cells, and the high expression of TNK2 in colon cancer might play a vital role in tumor prognosis. *TNK2* and miR-125a-3p could also serve as biomarkers and potential prognostic markers in colon cancer.

## Data Availability Statement

The original contributions presented in the study are included in the article/supplementary material, further inquiries can be directed to the corresponding author.

## Author Contributions

SL performed the majority of experiments and wrote the manuscript. PH designed the study. YH and YM provided vital reagents and analytical tools. XL, YL, and BK analyzed the data and were also involved in editing the manuscript. All authors contributed to the article and approved the submitted version.

## Consent For Publication

All authors gave final approval of the version to be published and agreed to be accountable for all aspects of the work.

## Conflict of Interest

The authors declare that the research was conducted in the absence of any commercial or financial relationships that could be construed as a potential conflict of interest.

## Publisher's Note

All claims expressed in this article are solely those of the authors and do not necessarily represent those of their affiliated organizations, or those of the publisher, the editors and the reviewers. Any product that may be evaluated in this article, or claim that may be made by its manufacturer, is not guaranteed or endorsed by the publisher.
